# The retail food environment and its association with body mass index in Mexico

**DOI:** 10.1038/s41366-021-00760-2

**Published:** 2021-02-17

**Authors:** Elisa Pineda, Eric J. Brunner, Clare H. Llewellyn, Jennifer S. Mindell

**Affiliations:** 1 Research Department of Epidemiology and Public Health, University College London, London, UK; 2 Centre for Health Economics & Policy Innovation (CHEPI), Imperial College Business School, London, UK; 3 School of Public Health, Imperial College London, London, UK; 4 Research Department of Behavioural Science and Health, University College London, London, UK

**Keywords:** Nutrition, Obesity, Risk factors, Epidemiology, Health policy

## Abstract

**Background/Objective:**

Mexico has one of the highest rates of obesity and overweight worldwide, affecting 75% of the population. The country has experienced a dietary and food retail transition involving increased availability of high-calorie-dense foods and beverages. This study aimed to assess the relationship between the retail food environment and body mass index (BMI) in Mexico.

**Subjects/Methods:**

Geographical and food outlet data were obtained from official statistics; anthropometric measurements and socioeconomic characteristics of adult participants (*N* = 22,219) came from the nationally representative 2012 National Health and Nutrition Survey (ENSANUT). Densities (store count/census tract area (CTA)) of convenience stores, restaurants, fast-food restaurants, supermarkets and fruit and vegetable stores were calculated. The association of retail food environment variables, sociodemographic data and BMI was tested using multilevel linear regression models.

**Results:**

Convenience store density was high (mean (SD) = 50.0 (36.9)/CTA) compared with other food outlets in Mexico. A unit increase in density of convenience stores was associated with a 0.003 kg/m^2^ (95% CI: 0.0006, 0.005, *p* = 0.011) increase in BMI, equivalent to 0.34 kg extra weight for an adult 1.60 m tall for every additional 10% store density increase (number of convenience stores per CTA (km^2^)). Metropolitan areas showed the highest density of food outlet concentration and the highest associations with BMI (*β* = 0.01, 95% CI: 0.004–0.01, *p* < 0.001). A 10% store density increase in these areas would represent a 1 kg increase in weight for an adult 1.60 m tall.

**Conclusions:**

Convenience store density was associated with higher mean BMI in Mexican adults. An excessive convenience store availability, that offers unhealthy food options, coupled with low access to healthy food resources or stores retailing healthy food, including fruits and vegetables, may increase the risk of higher BMI. This is the first study to assess the association of the retail food environment and BMI at a national level in Mexico.

## Introduction

The food environment—the collective physical, economic, policy and sociocultural surroundings, opportunities and conditions that influence people’s food and beverage choices and nutritional status—is recognised as a major determinant of health [1, 2] which can exert a major influence on obesity [3–7]. The community nutrition environment [8], or what we refer to as the retail food environment [9–11], encompasses the type, location and accessibility of food outlets. Retail food environment studies have mainly focused on high-income countries; however, there are a number of studies that have been conducted in low and middle-income countries (LMICs) [12–14], although very few have tested associations with obesity [15–20].

The North American Free Trade Agreement (NAFTA) came into force in 1994 to promote economic growth in the United States (US), Canada and Mexico [21]. Although the economic benefits of the treaty have been widely studied, its impact on health, particularly obesity, is unknown. NAFTA influenced the retail food environment by the introduction into Mexico of global fast-food outlet chains and an increased import of low-cost and ultra-processed foods [22–24]. The rise in obesity prevalence has accelerated worldwide in the last 30 years [25], particularly in Mexico, where measurement data from the 1988, 1999 and 2000 Nutrition and Health Surveys indicate a steep increase in obesity [26, 27], coinciding with the period when NAFTA was introduced [24]. Currently in Mexico, 75% of the population is affected by overweight or obesity [28].

Multiple factors, such as dietary intake, physical activity, the environment, socioeconomic position (SEP), deprivation, urbanicity and genetics, are associated with obesity [29, 30].

The worldwide rapid change in obesity prevalence cannot be explained by a single factor, such as changes in genes, individual-level psychosocial correlates of diet, or physical activity behaviours. Individualistic and single focused approaches to tackle obesity ignore the complex influences on dietary intake and obesity [31]; therefore, the solution to tackle obesity must lie in broader environmental, policy, and societal changes [32].

The rapid shift towards a greater obesity prevalence worldwide in the last few decades has been previously linked to changes in the retail food environment where energy-dense, nutrient-poor foods have become more widely available and accessible [33]. A high variation in body mass index (BMI) in the population and individual differences may reflect differential genetic susceptibility to the environment, with some individuals more susceptible to the modern ‘obesogenic’ retail food environment than others [34]. Therefore, along with a decline in physical activity and the industrialisation of food production, the large changes in the *mean* weight of the population suggests that the rapid changes in the retail food environment [24, 35] have greatly contributed to the obesity epidemic [33, 34, 36].

A study examining the retail food environment in a Mexican city indicated that communities in Mexico have an excessive access and exposure to calorie-dense foods and beverages [37]. The current retail food environment in Mexico has been greatly influenced by the US’ retail food environment, where individuals’ exposure to advertisements and proximity to energy-dense, nutrient-poor foods has increased [38]. The US population has seen a more than 50% increase in portion sizes in the past 20 years, equating to an additional 1,370 extra calories per day, if consumed daily [39]. All these exposures from the retail food environment encourage overconsumption, leading to obesity for many [38]. Acquiring low-nutrient-dense foods that are also calorie-dense has become more accessible and affordable in Mexico [24, 40], whilst healthy food is more expensive [41]. In 2012, the population in Mexico consumed a mean of 163 L of sugar-sweetened beverages (SSB) per person annually, equivalent to 0.5 L/day [42, 43]. As a consequence, Mexico was one of the first countries to introduce a SSB tax [42, 44, 45]. However, no food retail regulations have been introduced to improve healthy food availability at a national level [46, 47].

The aims of this study were to analyse the association between individual food outlet densities (food outlet count per census tract area (CTA)) and adult BMI in urban areas of Mexico, which are geographic areas with 2,500 inhabitants or more, and to establish which aspects of the retail food environment might be most obesogenic, in order to inform targeted policies regarding environmental regulations. We address the question of whether the retail food environment is a risk factor for higher BMI in middle-income countries such as Mexico.

## Subjects and methods

This study comprised secondary analysis of cross-sectional, population-based survey data of the spatial distribution of overweight and obesity across Mexico in conjunction with an analysis of the retail food environment and its association with BMI.

### Data sources and population sample

Sociodemographic and anthropometric data were obtained from the 2012 Mexican National Survey of Health and Nutrition (ENSANUT), which relies on measured anthropometric data and takes place every 6 years to asses dietary intake and health status of the general population [48]. Retail data were obtained from the 2014 economic census from the National Institute of Statistics, Geography and Informatics (INEGI, Spanish acronym) [49] (Table S1). The geographical areas of study were urban neighbourhoods and states in the country of Mexico. CTA was used as a proxy for neighbourhoods. A CTA in Mexico is defined as a geographic area formed by a set of blocks delimited by streets or identifiable pathways with land used for inhabiting, industrial or commercial services. Urban CTAs contain a population of ≥2,500 inhabitants [50]. There were 55,427 urban CTAs in this study, with a mean geometric area of 0.59 km^2^. The smallest CTA was 0.009 km^2^ and the largest 5.20 km^2^ (Table 1). For states, we focused on the 32 first-level administrative territorial entities in Mexico.

**Table 1:** Distribution of food outlets in urban census tract areas in Mexico.

Food outlet/area	Mean count (SD)	Min count	Max count	Mean density (SD)	Median density	Min density	Max density	p25	p50	p75	IQR	Number of CTA with no food outlets
Convenience stores	21.4 (14.0)	0	105	50.00 (36.9)	43.28 (36.85)	0	438	36.85	43.28	67.94	45.24	119
Fast-food outlets	2.0 (2.4)	0	30	4.85 (6.4)	2.82 (6.41)	0	71	0	2.82	7.04	7.04	7,704
Restaurants	9.9 (12.4)	0	111	23.22 (29.3)	14.09 (29.29)	0	262	4.13	14.09	32.92	28.79	2,715
Supermarkets	0.2 (0.5)	0	11	0.35 (1.2)	0.00 (1.16)	0	9	0	0	0	0	21,209
Fruit and vegetable stores	2.4 (9.3)	0	199	5.90 (22.7)	1.63 (22.74)	0	547	0	1.63	5.90	5.96	10,145
CTA geometric area (km^2^)^a^	0.5 (0.6)	0.0002	36	NA	NA	NA	NA	NA	NA	NA	NA	NA

Min: minimum count or density of food outlets per census tract area, Max: maximum count or density of food outlets per census tract area, NA: not applicable because it is a geographic area.

Count of food outlets is per census tract area (CTA) in km^2^. Density units are food outlet count in urban CTAs where the National Health and Nutrition Survey in Mexico (2012 ENSANUT) participants live.

*SD* standarad deviation, *p25* 25th percentile, *p50* 50th percentile, *p75* 75th percentile, *IQR* Interquartile range = p75 − p25

^a^*CTA*: Census tract area. Total number of urban CTAs = 55,427.

This study excluded data from women who were pregnant, survey participants <18 years of age, and participants without a valid, measured weight and height. Participants with BMI values of >3 standard deviations from the mean were excluded (<15 kg/m^2^ and >58 kg/m^2^) in case of possible underlying illnesses, eating disorders or implausible values. The adult population sample was 37,969. After excluding individuals living in rural areas, 22,219 individuals were kept as the analytical sample.

### Demographic, socioeconomic, and anthropometric variables

Sociodemographic, economic, health, anthropometric and variables that influenced the selection of individuals into the health survey (urbanicity, health service user, food assistance programme participation) were used in the analytical models to account for selection bias. Height, weight and waist circumference were measured by trained 2012 ENSANUT personnel. BMI was calculated as weight (kg) divided by height squared (m^2^). Participants with a BMI ≥ 30 kg/m^2^ were classified as having obesity. Disease risk, relative to normal weight and waist circumference, was considered to increase if waist circumference was >102 cm in men or >88 cm in women [51].

Data from the 2012 ENSANUT survey used to account for confounders in models included: age, gender, SEP, physical activity, car ownership, neighbourhood deprivation level, participation in a food assistance programme and health insurance. Physical activity and car ownership data were available for a subsample of the population (*n* = 10,587 and *n* = 8,635, respectively).

### Food retail classification

In 2014, INEGI carried out an economic census and digital georeferencing of establishments that produce goods, market merchandise and provide services, such as food retailers [50]. Through ground truthing (on-site verification) and using a survey and digital georeferencing, INEGI collected data on type of outlet, type of food provided, number of employees (which gave an approximation of the size of the food outlet), website, telephone number and location characteristics of the retailer. According to this, food retailers were classified by INEGI as restaurants, supermarkets, fruit and vegetable stores, chain type of convenience stores and non-chain type of convenience stores. We relied on the data collection from INEGI, regarding the characteristics and type of food provided in each food outlet, to generate a more specific food outlet classification that related to the availability of ultra-processed foods, fruits and vegetables and other food options. In store-assessment was not carried out. When there was doubt about the classification of the food outlet, we assessed and classified the food outlet according to the main food products that were sold and available on the food outlet’s website. For this study, food outlets, including informal and mobile food carts, that specialised in pizzas, hamburgers, hotdogs, and fried chicken were classified as fast-food outlets. Outlets that mainly sold SSBs and unhealthy snacks were classified as convenience stores. We assumed that all convenience stores and fast-food outlets sold mainly SSBs, snacks and ultra-processed foods. Food outlets with an á la carte menu, that included healthy food alternatives with sitting options available, were classified as restaurants. Mega-supermarkets and grocery stores, which offered greater food options than convenience stores, including fruits and vegetables, were classified as supermarkets. Outlets that specialised in fruits and/or vegetables were classified as fruit and vegetable stores.

### Geocoding of individuals and food outlets

Participants from the 2012 ENSANUT were geocoded to the geographic centroid of their urban CTA with ArcGIS 10.2.2 (ESRI, Redlands, CA). Exact address was unavailable due to data protection. Geographic coordinates of food outlets were tracked using a mobile computing device by INEGI and obtained from a national, governmental and publicly available dataset from INEGI, 2014 [52]. On-site verification, known as ground truthing [53] of nine geographic area samples was undertaken to verify the geolocation, existence, and type of food outlet in January 2016. Urban CTAs were grouped into a single shapefile, which was spatially merged with geolocation and sociodemographic characteristics of participants and food outlets.

### Geographical analyses

Density was calculated considering the count of food outlets per CTA divided by the CTA’s geographic area (km^2^). Density calculations and distribution were mapped through ArcGIS 10.2.2. Individual food outlet densities were calculated for convenience stores, restaurants, fast-food outlets, supermarkets and fruit and vegetable stores.

### Statistical analyses

We hypothesised that higher individual densities of supermarkets, fruit and vegetable stores and restaurants would be linked to a lower BMI whilst higher densities of fast-food outlets and convenience stores would be associated with a higher BMI. Three statistical models were computed using multilevel linear regression considering state (Models A and C) or CTA (Model B) as a second-level random effect to account for nesting of individuals within CTA or states; a fourth model (Model D), accounted for selection bias and was computed using linear regression. The models were constructed after drawing the postulated relationship of variables through directed acyclic graphs (DAG) [54], which captures the dependence structure of multiple variables and allow more robust conclusions about the direction of causation. While causality cannot be fully determined from cross-sectional data, DAGs indicate the relationships providing the best fit [55]. Multicollinearity was measured by variance inflation factors, which did not exceed the value of 4.0 for any of the included variables and were therefore all included in the models. Likelihood ratio and interclass correlation were tested. These tests helped select the best number of levels and variables to include in the models. The analysed data met the assumptions of the tests (e.g., normal distribution).

The dependent variable was BMI (continuous variable). To estimate variation within BMI groups, mean BMI and point estimates with confidence intervals were determined. Age, gender, household SEP (quintiles), physical activity (inactive, moderately active, active), car ownership (owns, does not own a car), region (north, centre, metropolitan, south), neighbourhood deprivation (low, high), urbanicity (rural (excluded from analyses), urban, metropolitan), food assistance programme (participates, does not) and health insurance (covered, not covered) were the independent variables tested as potential confounders through the different models. Bonferroni corrections were undertaken for all models. Data and code availability are available upon request. The variables included in each model were as follows:

**Model A**: Age, gender, and household SEP, with state as a second level.

**Model B**: Model A + physical activity, car ownership, neighbourhood deprivation and urbanicity level, food assistance programmes and health insurance, with CTA as a second level.

**Model C**: Model A + neighbourhood deprivation and urbanicity level, with state as a second level.

**Model D**: Model A + neighbourhood deprivation and urbanicity level, food assistance programme participation and health insurance attainment (to account for selection bias), linear regression.

**Mutually adjusted model**: Model A + all food outlet types, with state as a second level.

### BMI and its association with SEP

The SEP indicator was obtained from the 2012 ENSANUT [48, 56]. It was generated by the survey data owners by imputing deciles of income level to the households surveyed on the 2012 ENSANUT, using demographic and socioeconomic characteristics, including characteristics of the head of the family, sociodemographic structure, characteristics of the home, household goods, family consumption patterns and characteristics of the geographical area of residence based on the 2010 National Income and Expenditure Survey [57]. As a validation, distribution of different household characteristics related to SEP was described by predicted decile. Deciles were compared with a measure of poverty to create quintiles which were then assigned equally to each household member. A lower quintile indicates lower income whilst a higher quintile indicates a higher income [56].

To understand further the relationship of the retail food environment and BMI, the association between SEP and food outlet density was tested through the stratification of models by SEP quintiles. The association of SEP and the retail food environment was also tested using a two-level multinomial logistic regression with random effects to assess the role of socioeconomic aspects of the environment and how they could potentially influence the risk of higher BMI in urban CTAs in Mexico.

### Sensitivity analyses

Analyses were re-analysed with waist circumference as the main outcome to verify the relationship of adiposity and the retail food environment. Waist circumference (indicating abdominal fat) is more closely related to adverse health outcomes than BMI is [58, 59], but the available sample size was smaller. Interaction tests between SEP and food outlet density were undertaken (Supplementary Fig. S1).

Characteristics of the survey design—clustering, stratification, and finite-population corrections—were accounted for to obtain appropriate point estimates and standard errors when using the 2012 ENSANUT survey. Survey design was used in all descriptive and regression analyses and weights were accounted for in multilevel linear regression models. All the statistical analyses in this study were conducted using STATA, Statistical Software: Release 15, College Station, TX: StataCorp LP.

## Results

### Descriptive analyses

Table 2 shows the distribution of the study participants by BMI classification and sociodemographic, economic and health variables used in the analyses. Mean BMI and point estimates for linear combinations with confidence intervals indicate that women and 45 to 54-year-old individuals were more likely to develop obesity. Compared with active individuals, those who were inactive or moderately active were more likely to be individuals with obesity. Individuals with lowest and second lowest household SEP were also more likely to increase their risk of obesity compared with the highest household SEP individuals. Only 23% owned a car. For comparison, 44% of households in Mexico own at least one car [60]. The majority (39%) of the studied sample lived in the south of the country, in a CTA with a high deprivation level (37%) and in urban or metropolitan areas (64%).

**Table 2:** Sociodemographic, economic, health and anthropometric characteristics of Mexican adults (*n* = 37,969) aged 18+ inhabiting urban areas of Mexico; data from the nationally representative, cross-sectional, 2012 ENSANUT^a^.

Variable	Underweight^b^	Healthy weight^b^	Overweight^b^	Obese^b^	Total *N* (%)	Mean BMI (kg/m^2^) (95% CI)	*β* (95% CI)	*p* value
	*N* (%)	*N* (%)	*N* (%)	*N* (%)				
All	618 (100)	10,772 (100)	14,122 (100)	12,457 (100)	37,969 (100)			
Gender								
Men	259 (42)	5,324 (49)	6,671 (47)	4,421 (35)	16,675 (44)	27.37 (27.26, 27.48)	Ref	
Women	359 (58)	5,448 (51)	7,451 (53)	8,036 (64)	21,294 (56)	28.63 (28.52, 28.74)	1.25 (1.11, 1.40)	<0.001
Age								
18–24	298 (48)	3,306 (31)	1,725 (12)	1,067 (9)	6,396 (17)	25.29 (25.12, 25.47)	Ref	
25–34	87 (14)	2,194 (20)	2,738 (19)	2,197 (18)	7,216 (19)	27.86 (27.70, 28.03)	−2.57 (−2.80, −2.33)	<0.001
35–44	48 (8)	1,623 (15)	3,512 (25)	3,391 (27)	8,574 (23)	29.29 (29.14, 29.43)	−3.99 (−4.21, −3.78)	<0.001
45–54	30 (5)	1,142 (11)	2,505 (18)	2,739 (22)	6,416 (17)	29.59 (29.42, 29.77)	−4.30 (−4.53, −4.06)	<0.001
55–64	33 (5)	920 (9)	1,780 (13)	1,734 (14)	4,467 (12)	29.13 (28.93, 29.32)	−3.83 (−4.08, −3.58)	<0.001
65+	122 (20)	1,587 (15)	1,862 (13)	1,329 (11)	4,900 (13)	27.41 (27.21, 27.60)	−2.11 (−2.36, −1.86)	<0.001
Physical activity								
Active	29 (5)	460 (4)	619 (4)	670 (5)	1,778 (17)	28.00 (27.82, 28.17)	Ref	
Moderately active	19 (3)	306 (3)	390 (3)	417 (3)	1,132 (11)	28.57 (28.10, 29.04)	−0.57 (−1.07, −0.07)	0.024
Inactive	96 (15)	2,260 (21)	2,877 (20)	2,451 (20)	7,677 (20)	28.57 (28.23, 28.90)	−0.57 (−0.96, −0.17)	0.005
Missing	474 (77)	7,746 (72)	10,243 (72)	8,919 (72)	27,382 (52)	28.03 (27.94, 28.12)	−0.03 (−0.22, 0.15)	0.732
Household SEP^c^								
Highest	141 (32)	2,575 (28)	2,735 (20)	2,008 (17)	7,459 (21)	27.28 (27.09, 27.46)	Ref	
Second highest	86 (20)	1,841 (20)	2,758 (20)	2,510 (21)	7,195 (20)	28.45 (28.28, 28.62)	−0.13 (−0.36, 0.09)	0.246
Middle	71 (16)	1,692 (18)	2,751 (20)	2,554 (21)	7,068 (20)	28.52 (28.34, 28.70)	0.16 (−0.08, 0.41)	0.188
Second lowest	80 (18)	1,529 (17)	2,606 (19)	2,552 (21)	6,767 (19)	28.82 (28.66, 28.98)	0.24 (−0.0005, 0.47)	0.051
Lowest	58 (13)	1,510 (16)	2,573 (19)	2,439 (20)	6,580 (19)	28.69 (28.52, 28.86)	1.41 (1.16, 1.66)	<0.001
Car ownership								
Owns a car	101 (16)	2,189 (20)	3,334 (24)	3,011 (24)	8,635 (23)	28.24 (28.24, 28.53)	Ref	
Does not own a car	516 (83)	8,575 (80)	10,775 (76)	9,437 (76)	29,303 (77)	27.96 (27.87, 28.06)	0.42 (0.25, 0.59)	<0.001
Missing	1 (0)	8 (0.01)	13 (0.02)	9 (0.02)	31 (0.1)	27.54 (25.66, 29.43)	0.84 (−1.05, 2.72)	0.383
Region								
South	153 (35)	3,557 (39)	5,393 (40)	4,652 (39)	13,755 (39)	28.27 (28.13, 28.41)	Ref	
North	116 (27)	1,988 (22)	3,040 (23)	3,241 (27)	8,385 (24)	28.89 (28.72, 29.05)	−0.62 (−0.83, −0.40)	<0.001
Centre	162 (37)	3,513 (38)	4,822 (36)	4,019 (33)	12,516 (36)	28.12 (27.98, 28.26)	0.15 (−0.042, 0.35)	0.124
Metropolitan area	5 (1)	89 (1)	168 (1)	151 (1)	413 (1)	28.65 (28.04, 29.26)	−0.38 (−1.04, 0.24)	0.232
Deprivation								
Low	376 (61)	6,336 (59)	8,833 (65)	8,259 (66)	23,804 (63)	28.23 (28.13, 28.33)	Ref	
High	242 (39)	4,436 (41)	5,289 (37)	4,198 (34)	14,165 (37)	27.47 (27.35, 27.60)	0.80 (0.60, 0.92)	<0.001
Urbanicity								
Rural	242 (39)	4,365 (41)	4,993 (35)	3,801 (31)	13,401 (35)	27.31 (27.17, 27.46)	Ref	
Urban	114 (18)	2,156 (20)	3,153 (22)	3,030 (24)	8,453 (22)	28.45, (28.27, 28.63)	−1.14 (−1.37, −0.91)	<0.001
Metropolitan	262 (42)	4,251 (39)	5,976 (42)	5,626 (45)	16,115 (42)	28.30 (28.18, 28.42)	−0.99 (−1.73, −0.80)	<0.001
Food programme								
Participates	461 (75)	8,161 (76)	10,941 (77)	9,620 (77)	29,183 (77)	28.10 (28.01, 28.19)	Ref	
Does not participate	157 (75)	2,611 (76)	3,181 (77)	2,837 (23)	8,786 (23)	27.92 (27.74, 28.09)	0.18 (0.0001, 0.36)	0.05
Health service								
Covered	191 (31)	2,709 (25)	2,718 (19)	2,338 (19)	7,956 (21)	27.53 (27.37, 27.69)	Ref	
Not covered	427 (69)	8,063 (75)	11,404 (81)	10,119 (81)	30,013 (79)	28.23 (28.14, 28.32)	−0.69 (−0.087, −0.52)	<0.001

Mean BMI (kg/m^2^) (95% CI): mean body mass index with 95% confidence interval by category using survey design.

*β* (95% CI): beta coefficient and 95% confidence interval represent point estimate for linear combinations of coefficients. Each variable is compared with the reference category.

^a^ENSANUT: National Health and Nutrition Survey in Mexico.

^b^Body mass index (BMI): underweight: 15 to <18.5 kg/m^2^; healthy weight: ≥18.5 to <25 kg/m^2^; overweight: 25 to <30 kg/m^2^; obese: ≥30 kg/m^2^.

^c^Quintiles.

In terms of the retail food environment, convenience stores had the highest count and density of food outlets per CTA (km^2^) (Table 1). CTAs in metropolitan areas, had the highest concentration, with up to 105 convenience stores per CTA, equivalent to a density of 438/CTA km^2^ density (Table 1). After convenience stores, restaurants, fruit and vegetable stores, fast-food outlets and supermarkets followed in order from higher-to-lower count of stores and density (Table 1). However, even though it appears that there is a high density of fruit and vegetable stores, in Mexico, fruit and vegetable stores are more available in the South of than in the North of Mexico (Supplementary Fig. S2). Urban CTAs had a mean area of 0.46 ± 0.56 km^2^—the smallest CTA being 0.0002 km^2^ and the largest 36.41 km^2^ (Table 1).

Convenience stores were the most widely available food outlets within neighbourhoods in Mexico. The highest concentration of convenience stores was found in metropolitan areas. Fruit and vegetable stores were the least available per CTA, followed by fast-food outlets and supermarkets (Table 1 and Supplementary Fig. S2). Many urban CTAs in Mexico did not contain a fruit and vegetable store (*n* = 10,145 CTAs without fruit and vegetable store, 42%) or supermarket (*n* = 21,209 CTAs without supermarket, 88%), whereas only 119 CTAs (0.5%) did not contain a convenience store (Supplementary Fig. S2).

### Associations between individual food outlet densities and BMI

Results for individual food outlet densities and their relationship with BMI are shown in Table 3. A higher availability of convenience stores in neighbourhoods was associated with a higher mean BMI in Model A (*β* = 0.003, 95% CI: 0.0006, 0.005, *p* = 0.011), Model C (*β* = 0.003, 95% CI: 0.0009, 0.005, *p* = 0.006), Model D (*β* = 0.003, 95% CI: 0.0001, 0.005, *p* = 0.041) and the mutually adjusted model (*β* = 0.003, 95% CI: 0.001, 0.006, *p* = 0.006). Model A had the highest (best) intraclass correlation coefficient, recommended for the assessment of the reliability of measurement scales [61], (0.11, SE: 0.02, 95% CI: 0.07, 0.17). According to the results of these models, a 10% convenience store increase in a CTA with a high concentration of convenience stores, referred to maximum density in Table 1, would be equivalent to a 0.13 kg weight increase in an adult 1.60 m tall. These associations remained despite adjusting for age, gender, and SEP in Model A; deprivation, SEP, urbanicity of CTA in Model C; after accounting for sample selection bias in model D, and after a Bonferroni correction.

**Table 3:** Multivariate associations between food outlet density per census tract area (km^2^) in urban areas of Mexico and BMI^a^ of Mexican adults from the 2012 ENSANUT^b^.

Food outlet density	Model A		Model B		Model C		Model D		Mutually adjusted model	
	*N* = 22,219		*N* = 6,068		*N* = 22,219		*N* = 22,219		*N* = 22,219	
	*β* (95% CI)	*p* value	*β* (95% CI)	*p* value	*β* (95% CI)	*p* value	*β* (95% CI)	*p* value	*β* (95% CI)	*p* value
Convenience stores	**0.003 (0.0006, 0.005)**	**0.011**	0.0002 (−0.004, 0.004)	0.924	**0.003 (0.0009, 0.005)**	**0.006**	**0.003 (0.0001, 0.005)**	**0.041**	**0.003 (0.001, 0.006)**	**0.006**
Fast-food outlets	0.002 (−0.009, 0.013)	0.716	0.016 (−0.007, 0.040)	0.180	0.003 (−0.009, 0.015)	0.614	0.007 (−0.008, 0.02)	0.387	0.0003 (−0.014, 0.015)	0.971
Restaurants	0.00007 (−0.002, 0.002)	0.957	−0.002 (−0.007, 0.003)	0.444	0.0002 (−0.0025, 0.003)	0.735	−0.002 (−0.005, 0.002)	0.384	−0.001 (−0.005, 0.002)	0.451
Supermarkets	−0.035 (−0.096, 0.026)	0.259	−0.017 (−0.14, 0.11)	0.788	−0.035 (−0.100, 0.027)	0.270	−0.06 (−0.14, 0.02)	0.155	−0.033 (−0.10, 0.03)	0.319
Fruit and vegetable stores	0.0004 (−003, 0.004)	0.793	−0.002 (−0.01, 0.007)	0.689	0.0004 (−0.003, 0.004)	0.786	0.001 (−0.003, 0.006)	0.584	−0.0001 (−0.004, 0.004)	0.939

All results indicate coefficients (*β*) and 95% confidence interval (CI) in parenthesis. *β* represents the increase of BMI in kg/m^2^ per every unit increase of food outlet density (count of food outlets per census tract area in km^2^). Bold values indicate statistical significance at the *p* < 0.05 level.

Model A: age, gender and socioeconomic position.

Model B: Model A + physical activity, car ownership, neighbourhood deprivation and urbanicity level, food assistance programmes, health insurance, and socioeconomic position, CTA (2nd level).

Model C: Model A + neighbourhood deprivation and urbanicity level.

Model D: Model A + neighbourhood deprivation and urbanicity level, food assistance programmes and health insurance.

Mutually adjusted models: Model A + all food outlets.

^a^*BMI* body mass index.

^b^*ENSANUT* National Health and Nutrition Survey in Mexico.

Although the point estimates were similar, no statistically significant associations were found in Model B. Supermarkets, fruit and vegetable stores and restaurants pointed in the direction of being protective against a higher BMI, whilst fast-food outlets indicated higher odds of being associated with a higher BMI; however, no statistically significant results were found for any of these food outlets.

### Association of food outlets with BMI by region

When looking at the association of food outlets with BMI by region, metropolitan areas showed the highest density of food outlets and the highest associations with higher BMI values. A high density of convenience stores was associated with a higher BMI (*β* = 0.01, 95% CI: 0.004–0.01, *p* < 0.001) (Table 4). Considering that metropolitan areas have a maximum density of 438 convenience stores per CTA (km^2^), a 10% increase in convenience stores would represent a risk of 1 kg increase in weight for a person weighting 65 kg and 1.60 m high, for individuals inhabiting metropolitan areas with a maximum convenience store density. This would represent a BMI increase from 25.4 to 25.8 kg/m^2^.

**Table 4:** Multivariate associations between food outlet density per census tract area (km^2^) in urban areas of Mexico and BMI^a^ of Mexican adults from the 2012 ENSANUT^b^, stratified by region.

Food outlets	Region							
	North		Centre		Metropolitan		South	
	*N* = 6,412		*N* = 7,659		*N* = 413		*N* = 7,735	
	*β* (95% CI)	*p* value	*β* (95% CI)	*p* value	*β* (95% CI)	*p* value	*β* (95% CI)	*p* value
Convenience stores	−0.002 (−0.008, 0.003)	0.361	0.003 (−0.0004, 0.007)	0.078	**0.01** (**0.004, 0.01)**	**<0.001**	0.003 (−0.0003, 0.006)	0.078
Fast-food outlets	−0.01 (−0.04, 0.01)	0.319	−0.005 (−0.025, 0.01)	0.631	0.001 (−0.10, 0.10)	0.984	**0.02** (**0.002, 0.04)**	**0.027**
Restaurants	−0.008 (−0.02, 0.002)	0.109	−0.004 (0.008, 0.0006)	0.094	**0.02** (**0.004, 0.03)**	**0.013**	**0.004** (**0.0003, 0.007)**	**0.030**
Supermarkets	−0.02 (−0.13, 0.09)	0.692	−0.06 (−0.16, 0.04)	0.220	0.17 (−0.07, 0.42)	0.165	−0.03 (−0.15, 0.09)	0.619
Fruit and vegetable stores	−0.03 (−0.08, 0.01)	0.173	**−0.01** (**−0.03, −0.003)**	**0.019**	**0.01** (**0.002, 0.02)**	**0.012**	0.0005 (0.003, 0.004)	0.771

All results indicate coefficients (*β*) and 95% confidence interval (CI) in parenthesis. *β* represents the increase of BMI in kg/m^2^ per every unit increase of food outlet density (count of food outlets per census tract area in km^2^). Bold values indicate statistical significance at the *p* < 0.05 level.

Model A: age, sex and socioeconomic position.

^a^*BMI* body mass index.

^b^*ENSANUT* National Health and Nutrition Survey in Mexico.

### SEP and retail food environment

Results showed that convenience stores were more likely to be most densely concentrated around the second highest, second lowest and middle SEP households but tended to be least densely concentrated around the lowest or highest SEP households. Supermarkets, fruit and vegetable stores, restaurants and fast-food restaurants tended to be less available in the vicinity of the lowest SEP households (Supplementary Table S2).

When looking at the association of food outlet density and BMI stratified by SEP (Supplementary Table S3), the second lowest SEP households had a greater exposure to convenience stores and were more likely to be affected by a higher BMI than those of the highest and lowest SEP households (Model A: *β* = 0.006, 95% CI: 0.001, 0.001, *p* = 0.026; Model B: *β* = 0.006, 95% CI: 0.0006, 0.01, *p* = 0.029; Model C: *β* = 0.006, 95% CI: 0.001, 0.01, *p* = 0.19). For every 10% increase in convenience store density in the second lowest SEP households, this would represent a weight increase of 0.07 kg in a person of 65 kg and 1.60 m high when considering the median of convenience stores density (43 convenience stores per CTA km^2^) (Table 1).

Survey participants from the second lowest SEP households who were more exposed to a high level of restaurants had a higher BMI than those of other SEP (Model A: *β* = 0.01, 95% CI: 0.0006, 0.001, *p* = 0.029; Model B: *β* = 0.008, 95% CI: 0.0079, 0.001, *p* = 0.03; Model C: *β* = 0.008, 95% CI: 0.0004, 0.01, *p* = 0.038). Those of higher SEP, with more expendable income and greater supermarket availability, had lower BMI levels (Model B: *β* = −0.006, 95% CI: −0.01, −0.001, *p* = 0.009). No statistically significant findings were observed for other food outlets and BMI when stratifying by SEP (Table S3).

### Sensitivity analyses

Analyses with waist circumference as the outcome showed that a higher density of convenience stores was associated with a higher waist circumference for Model A (*β* = 0.005, 95% CI: 0003, 0.01, *p* = 0.037); Model C (*β* = 0.006, 95% CI: 0001, 0.01, *p* = 0.019); and the mutually adjusted model (*β* = 0.009, 95% CI: 003, 0.14, *p* = 0.003) (Table S4). As an example of the effect size, for model A, for every 10% increase in convenience store density, this association would be equivalent to a 0.22 cm waist circumference increase for a person with a 100 cm waist circumference. The effect by SEP on BMI changed by −0.057 kg/m^2^ (*β* = −0.057, 95% CI: −0.11, −0.004, *p* = 0.036) for lower income populations who are exposed to restaurant availability, which indicates that lower income households are less likely to have access to restaurants in their neighbourhood compared with other SEP populations. All other interactions were not statistically significant (Supplementary Fig. S1).

To address a potential false positive, or type 1 error, when making inferences about the association for the retail food environment and BMI, we carried out our statistical models with robust standard errors. Supplementary Table S5 shows all models that tested the association of the retail food environment and BMI accounting for robust standard errors. Results showed that there was no violation of the constant variance assumption, and we observed a repeated pattern in this study, where a high density of convenience stores is associated to higher BMIs.

## Discussion

In this study, we examined the relationship between the retail food environment and BMI in Mexico. Food outlet density was used as a measure of retail food environment. We found that retail food environments with higher densities of convenience stores were significantly associated with higher BMIs, even after adjustment for diverse sociodemographic variables. As an example of the potential implications, the results of Model A indicate that for every additional convenience store per CTA, BMI is 0.003 kg/m^2^ higher; equivalent to a 0.34 kg weight increase for a person weighing 65 kg and 1.60 m tall when considering a 10% density increase of a maximum density exposure of 438 convenience stores per CTA (km^2^) (Table 1). A higher density of convenience stores was significantly associated with a higher BMI in all adjusted models, except Model B (Table 3). Model B was adjusted for physical activity and car ownership, data that were available for only a sub-section of participants, leading to a reduced analytical sample (*n* = 6,068), limiting the statistical power of the model to find a true association (Table 3). However, it is plausible that being physically active could, to some extent, offset the additional energy intake resulting from an increased access to convenience stores [62]. In addition, having a car would enable the neighbourhood inhabitants to travel further to obtain food [63] so the result may indicate that the BMI of people who own a car is truly not associated with the density of convenience stores in their own neighbourhoods. People who own a car may be less likely to depend on local convenience stores as their main source of food shopping [63–65].

Convenience stores were the most highly prevalent store type in CTAs (Table 1). They could represent a risk for higher BMI because of their substantial offer of beverages with a high sugar content, fast-food and snacks with low nutritional value and a high content of saturated fat, salt and calories coupled with a low availability of fruits and vegetables if any [65–67]. Having a high density of this type of food outlet within neighbourhoods in Mexico can represent an increased risk for higher BMIs in the population.

When mutually adjusting for all food outlets, convenience stores remained a high-risk factor for higher BMI. The importance of this model is that it accounts for all the studied food outlets within the same geographic area and can therefore provide a better estimate of the influence of a food outlet (Table 3). The null findings for the other food stores could be linked to residual confounding. There are two main ways in which residual confounding could have affected the association of the retail food environment and BMI. First, there could have been additional confounding factors that were not considered in this study, such as dietary intake. Dietary intake could be a mediator of the association between convenience store density and BMI [68–72] Second, missing data could have impacted the association. The study’s sample was restricted to individuals who had a measure of the retail food environment and of height and weight, from which BMI was derived. Individuals who did not have a measure of obesity, lived in a rural area, or did not have a measure of the retail food environment were excluded from this study.

When assessing socioeconomic disparities, convenience stores were most accessible to second lowest, middle and second highest SEP households. However, extremely deprived (lowest SEP) and highest SEP households were less likely to have convenience stores in their neighbourhood. This can be explained by the different characteristics of these types of neighbourhoods. On the one hand, the highest SEP neighbourhoods have greater restrictions on commercial developments or tend to be further away from these types of establishments, whilst the most deprived areas, where the lowest SEP households tend to be located, lack infrastructure and services and have less availability of food outlets overall and may be more exposed to food insecurity [73, 74]. An association with disposable income is possible, where, due to neighbourhood disparities, the highest SEP group may live in areas where availability and accessibility of healthy foods is higher and can therefore shop at supermarkets and food outlets that offer healthy foods. The lowest SEP group generally has lower disposable income and may live in areas where there is lower food diversity and healthy food availability and accessibility, compared with high-income areas, and as observed by previous studies [75, 76]. However, as households increase their available income there tends to be a cross-over to a higher availability of food outlets—particularly convenience stores (Table S2). As disposable income begins to grow, there tends to be a greater exposure to convenience stores, and therefore a greater availability that goes along with an increased financial accessibility to unhealthy, ultra-processed foods and drinks [77]. As expected, this increased availability of convenience stores was associated with a higher BMI for the second lowest SEP households (Table S3). Therefore, due to a greater availability of convenience stores in neighbourhoods, which second lowest and middle SEP populations inhabit, these households may be exposed to a greater availability of the SSB and ultra-processed foods and appear to be more sensitive to the retail food environment, positioning this sector of the population at a greater risk for higher BMI [78].

Supporting this finding, a study by Pérez-Ferrer et al. indicated that household wealth can be an effect modifier in the association between education and obesity, mainly in women [79]. The study also indicated that as countries like Mexico develop economically, there tends to be a cross-over to higher rates of obesity among socially disadvantaged groups which may be explained by a nutrition transition which, as our study indicates, may have been led by this sub-population’s higher exposure to unhealthy food outlets. In Mexico, the nutrition transition and an increase in obesity coincided with the NAFTA agreement [24, 40], which may be related to growth in unhealthy food retail outlets [24].

The retail food retail environment and its association with BMI at a national level had not been assessed previously in any Latin American country. However, the findings of this study coincide with those from higher-income countries, where a greater availability of food stores equivalent to convenience stores in Mexico, which provide high accessibility of SSB and ultra-processed foods, has been linked with a higher risk of obesity [67, 80–83].

The findings presented here should be interpreted with caution. Among the limitations of this study the following issues are observed. The use of cross-sectional data, which does not permit causal inferences to be made. Retail food environments are dynamic, and a longitudinal design could help understand the effect of the retail food environment on obesity. Similarly, a natural experimental evaluation could have minimised potential bias and provide critical information about the impacts of food retail interventions on dietary intake [84] or obesity [85]. It is also possible that people with a predisposition towards obesity choose to live in areas with a higher density of convenience stores or that stores strategically open in places where people are more likely to consume from them. Therefore, the environment could be both a consequence of and a risk factor for higher BMI. There was also a 2-year difference between the health and geographic data that were used in this study. However, data verification two years later found the prevalence, position and type of food store was still accurate, suggesting little change over time. In addition, due to data confidentiality, BMI data were recorded at the centroid of the residents’ CTA as precise individual-level area data were not available due to data confidentiality.

CTA was used to calculate food outlet density as a proxy for individual’s food environment. Individuals often cross the boundaries of their residential area to access food which may underestimate food availability [86]; however, residents in impoverished areas may have limited capital resources, such as car ownership, making it feasible to assume that there may be greater reliance on proximal food sources [87]. CTA has been considered a gold standard for measuring food environment and has been used by various studies as the unit of analysis to study food environments [86, 88].

International franchises and local fast-food outlets were grouped together, considering that similar ultra-processed foods were offered in both type of establishments. No association was found between fast-food outlet density and BMI. It is possible that small locally owned fast-food outlets could be visited more, particularly in low-income neighbourhoods. Lastly, although informal food vendors were identified in the on-site food outlet location data-verification stage of sample areas, it is possible that some mobile food units, which represent an important influence on dietary intake in Mexico [89], may not have been included in the food outlet database used in this study. This could have been due to lack of registration compliance to sell food or because of the provision of a home address rather than the place of sale.

This study has several strengths. This is one of the few studies to have assessed the relationship between the retail food environments and BMI in a middle-income country. This study advances the existing literature of the retail food environment and its relationship with higher BMI and the application of geographical information systems. Other strengths include the use of measured data; the geographical location verification of food outlets; accounting for selection bias; and the use of a statistical method that was able to detect discrepancies between different geographical levels and account for clustering.

No country in the world has yet managed to reverse the rise in obesity prevalence in adults [90]. A combination of different approaches and interventions are required. Fiscal and regulatory approaches have been shown to be far more effective than interventions focused on changing individual behaviour [91]. Modifying and improving the environment so that healthier choices are more widely available and distributed could contribute towards the reduction of obesity prevalence and its health consequences. Fruitful approaches may involve regulating the number and opening times of convenience stores that are available; increasing the availability and accessibility of healthy foods in convenience stores; making fruit and vegetable stores more prominent and salient; increasing affordability of fruits and vegetables, shaping and guiding consumer’s choices into healthier choices (choice architecture).

To tackle obesity, retail food environment regulations are required in addition to measures that are isolated or focused on individuals, such as food labelling and SSB taxation. Regulating the retail food environment is not the sole answer to the obesity problem but is likely to be an important part of the solution.

## Conclusion

The obesogenic retail food environment in Mexico poses an increased risk of obesity for the population. This study showed that a higher density of convenience stores within neighbourhoods is associated with higher mean BMI. Policies and programmes implemented so far in Mexico, particularly the tax on SSB and widespread health promotion campaigns, have not been enough to halt and reverse the obesity epidemic. Policymakers should create additional policies and programmes that account for the social distribution of obesity prevalence and take action on the regulation of the retail food environment.

## Supplementary information

Supplementary Figure S1

Supplementary Figure S2

Supplementary Table S1

Supplementary Table S2

Supplementary Table S3

Supplementary Table S4

Supplementary Table S5

Supplementary figure legends
