# Supplementary figures and images for: The retail food environment and its association with body mass index in Mexico
Source: Int J Obes (Lond). 2021 Feb 17;45(6):1215–28. doi: 10.1038/s41366-021-00760-2 (PMC8159738; doi:10.1038/s41366-021-00760-2)

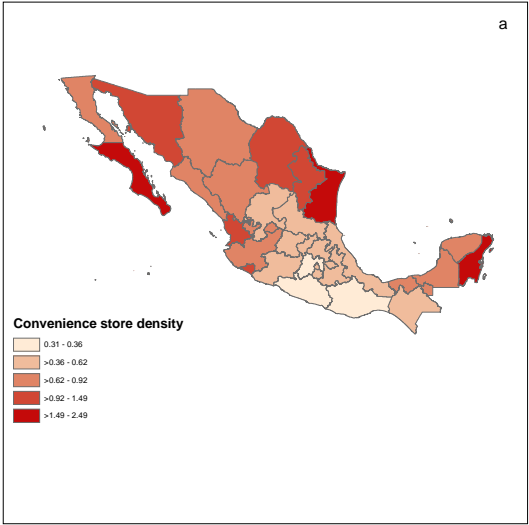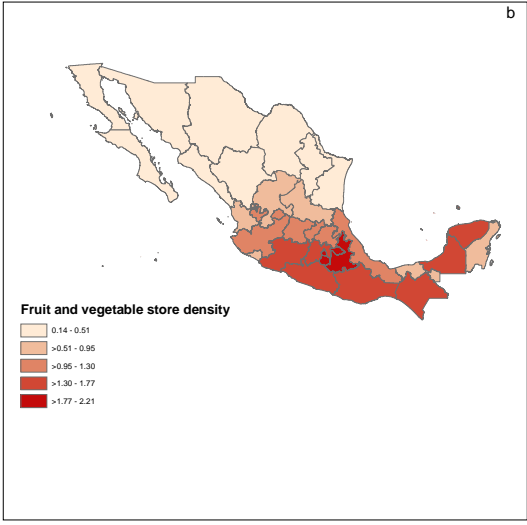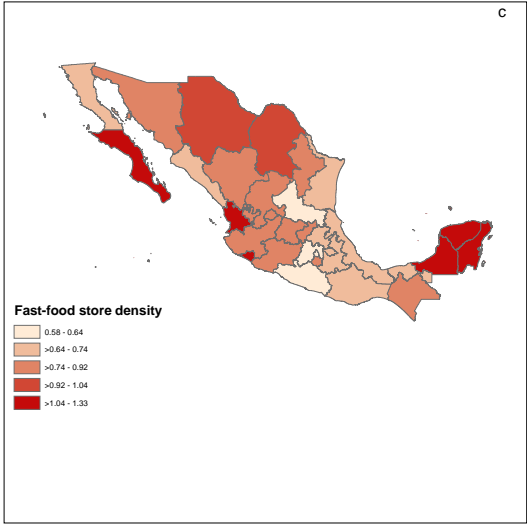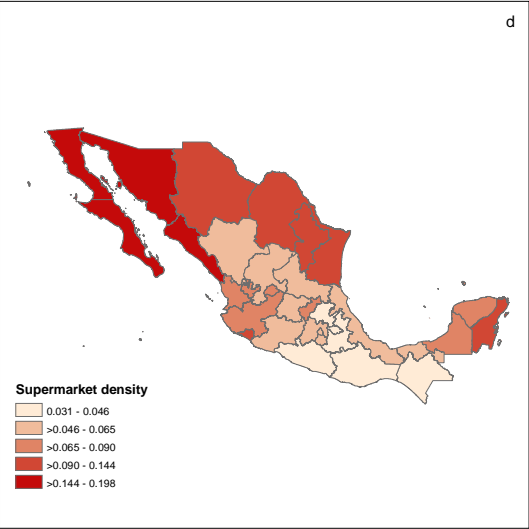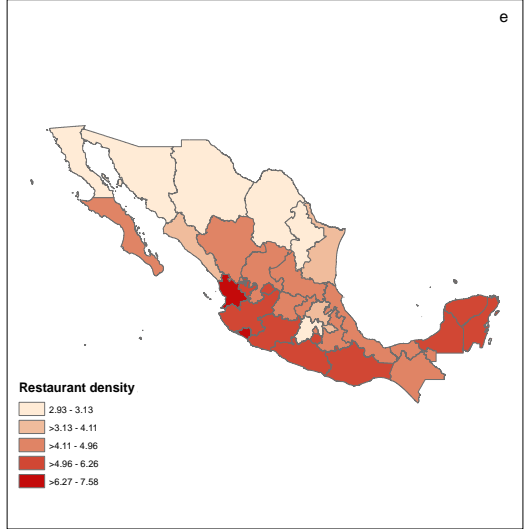

Supplement: Supplementary file 2 — Supplementary Figure S2 [file 41366_2021_760_MOESM2_ESM.pdf]
