# Supplementary material for: The retail food environment and its association with body mass index in Mexico
Source: Int J Obes (Lond). 2021 Feb 17;45(6):1215–28. doi: 10.1038/s41366-021-00760-2 (PMC8159738; doi:10.1038/s41366-021-00760-2)
Supplement: Supplementary file 8 — Supplementary figure legends [file 41366_2021_760_MOESM8_ESM.docx]

**Supplementary Figure S1. Interaction between food outlet density and socioeconomic position**

Coefficient of interaction indicates difference in the effect.

**Supplementary Figure S2. Food outlet density in Mexico**

a: Convenience store density per state; b: Fruit and vegetable store density per state; c: Fast-food store density per state; d: Supermarket density per sate; e: Restaurant density per state. Density was calculated considering the count of food outlets per CTA divided by the CTA’s geographic area (km^2^). Density calculations and distribution were mapped through ArcGIS 10.2.2. Food outlet density is shown at the state level for visualisation purposes.

**Supplementary Table S1. Health and geographic data sources used for this research**

a BMI: Body mass index; b CTA: Census tract area; c INEGI: National Institute of Statistics, Geography and Informatics (INEGI, Spanish acronym).

**Supplementary Table S2. Two-level multinomial logistic regression with random effects association between food outlet density and socioeconomic position of Mexican adults from the 2012 ENSANUT† and food outlet density per urban census tract areas (km^2^) in Mexico**

ENSANUT: National Health and Nutrition Survey in Mexico.

All results indicate coefficients (β) and 95% confidence interval (CI) in parenthesis. β represents the increase of BMI in kg/m^2^ per additional food outlet per km^2^. Two-level multinomial logistic regression with random effects was used to test the association of socioeconomic position and food outlet density.

Model A: Age, gender, and socioeconomic position.

Model B: Model A + physical activity, car ownership, neighbourhood deprivation, food assistance programmes, health insurance, and socioeconomic position, CTA (2nd level).

Model C: Model A + deprivation and urbanity of CTA.

Model D: Age, gender, neighbourhood deprivation, food assistance programmes, health insurance and socioeconomic position.

Mutually adjusted models: Model A + all food outlets.

**Supplementary Table S3. Association of food outlet density per census tract areas (km^2^) in Mexico and body mass index (BMI) stratified by quintile of socioeconomic position (SEP) of adults from the 2012 ENSANUT†**

ENSANUT: Mexican National Survey of Health and Nutrition

All β results indicate coefficients of the association of food outlet density and BMI, stratified by SEP, using multilevel linear regression. β represents the increase of BMI in kg/m^2^ per additional food outlet per km^2.^

Model A: Age, gender, and socioeconomic position.

**Supplementary Table S4. Multivariate associations between food store type and waist circumference**

All results indicate coefficients (β) and 95% confidence interval (CI) in parenthesis. β represents the increase of waist circumference (cm) per every unit increase of food outlet density.

Model A: Age, gender, and socioeconomic position.

Model B: Model A + physical activity, car ownership, neighbourhood deprivation, food assistance programmes, health insurance, and socioeconomic position, CTA (2nd level).

Model C: Model A + neighbourhood deprivation and urbanity level.

Model D: Model A + neighbourhood deprivation, food assistance programmes and health insurance.

Mutually adjusted models: Model A + all food outlets.

**Supplementary Table S5. Multivariate associations between food outlet density per census tract area (km^2^) in** **urban areas of Mexico and BMI† of Mexican adults from the 2012 ENSANUT†† using robust standard deviations**

^†^BMI: Body mass index, ^††^ENSANUT: National Health and Nutrition Survey in Mexico.

All results indicate coefficients (β) and 95% confidence interval (CI) in parenthesis. β represents the increase of BMI in kg/m^2^ per every unit increase of food outlet density (count of food outlets per census tract area in km^2^).

Model A: age, gender, and socioeconomic position.

Model B: Model A + physical activity, car ownership, neighbourhood deprivation, food assistance programmes, health insurance, and socioeconomic position, CTA (2nd level).

Model C: Model A + neighbourhood deprivation and urbanity level.

Model D: Model A + neighbourhood deprivation, neighbourhood urbanity level, food assistance programmes and health insurance.

Mutually adjusted models: Model A + all food outlets.
